# Peroxin Pex8 couples stress responses, antifungal tolerance, and virulence regulation in *Candida albicans*

**DOI:** 10.1128/aac.01662-25

**Published:** 2026-03-24

**Authors:** Bangsheng Xi, Yongqin Wu

**Affiliations:** 1 Department of Laboratory Medicine, The First Affiliated Hospital of Wannan Medical College (Yijishan Hospital of Wannan Medical College) 569222, Wuhu, Anhui, China; 2 Department of Laboratory Medicine, The First Affiliated Hospital of USTC, Division of Life Sciences and Medicine, University of Science and Technology of China 12652, Hefei, Anhui, China; 3 Core Unit of National Clinical Research Center for Laboratory Medicine, Hefei, Anhui, China; University Children's Hospital Münster, Münster, Germany

**Keywords:** *Candida albicans*, Pex8, antifungal tolerance, virulence

## Abstract

*Candida albicans*, a World Health Organization critical-priority fungal pathogen, represents the predominant cause of candidemia. Therapeutic failure is sometimes driven by antifungal tolerance, a phenotype distinct from resistance, whose underlying mechanisms remain incompletely defined. Here, we identify the peroxisomal protein Pex8 as a key regulator of tolerance to both azoles and amphotericin B. Although neither deletion nor overexpression of *PEX8* altered minimum inhibitory concentrations, both modifications significantly reduced drug tolerance, as demonstrated by reduced fractional growth (FoG_20_) and impaired survival under drug pressure. RNA-seq analysis showed that *PEX8* overexpression leads to downregulation of ergosterol biosynthesis genes and remodeling of stress-response pathways, suggesting a possible transcriptional basis for the loss of azole tolerance. Complementary lipidomic profiling demonstrated that *PEX8* genetic manipulations induce extensive membrane lipid remodeling, characterized by specific alterations in ceramide and lysophospholipid subclasses, thereby revealing an ergosterol-independent mechanism underlying amphotericin B tolerance attenuation. Phenotypically, *PEX8* overexpression attenuated serum-induced hyphal morphogenesis and reduced virulence in a *Galleria mellonella* infection model, consistent with downregulation of hyphal-associated and virulence-related genes. Our findings establish Pex8 as a central coordinator of oxidative stress adaptation, membrane homeostasis, filamentation, and pathogenicity, revealing a promising target for combating antifungal tolerance.

## INTRODUCTION

*Candida albicans* is a commensal–pathogenic yeast responsible for a broad range of human infections, spanning from mucosal conditions such as vulvovaginal candidiasis to life-threatening invasive candidiasis (1–3). Designated a critical priority fungal pathogen by the World Health Organization, it remains the leading species isolated from candidemia cases worldwide (4). Invasive *C. albicans* infections continue to pose a serious clinical challenge and are associated with high mortality rates, particularly among immunocompromised and critically ill patients, even with current antifungal treatments (1, 5). This persistent clinical impact, together with the growing problem of azole resistance, highlights the urgent need to elucidate the mechanisms driving treatment failure in *C. albicans* infections.

The establishment and progression of *C. albicans* infections are fundamentally supported by its diverse virulence arsenal and remarkable adaptability (6). A key virulence trait is its ability to switch between yeast and hyphal morphologies, a transition closely linked to pathogenicity (7). The yeast form facilitates dissemination, while hyphae are essential for tissue invasion, biofilm formation, and escape from phagocytic cells (7). Furthermore, *C. albicans* possesses an extensive repertoire of virulence factors, including adhesins (e.g., Als family proteins), hydrolases (e.g., Sap family proteases), and candidalysin, all of which are intricately regulated (8, 9). The ability to dynamically regulate these virulence determinants, particularly through morphological plasticity, establishes *C. albicans* as a formidable pathogen capable of causing persistent and often refractory infections (10). Therefore, exploring the regulatory genes that govern the yeast-to-hypha transition is crucial for gaining deeper insights into the pathogenic mechanisms of this fungus.

*C. albicans* generally exhibits high susceptibility to most antifungal agents *in vitro*, with resistance rates remaining relatively low (11, 12). When azole resistance occurs, it primarily arises from hotspot mutations in the *ERG11* gene or overexpression of efflux pump genes *CDR1* and *CDR2* (13). However, treatment failures, such as persistent candidemia, are not uncommon in clinical practice, even when *in vitro* susceptibility profiles indicate sensitivity (14, 15). Recent studies have revealed that antifungal tolerance, which is characterized by reduced *in vitro* susceptibility without an increase in minimum inhibitory concentration (MIC), contributes to persistent candidemia during azole therapy (16, 17). Research across *Candida* species has begun to elucidate specific mechanisms underlying this phenotype. In *C. albicans*, a calcineurin-dependent pathway involving the transcription factor Crz1 has been identified as a key determinant of fluconazole tolerance (18). In *Candida glabrata*, trehalose metabolism, governed by Tps2, critically mediates fluconazole tolerance by modulating cellular sterol composition (19). Similarly, in *Candida tropicalis*, tolerance to echinocandins has been linked to multicellular aggregation mediated by low expression of the transcription factor Ace2 (20). Beyond the *Candida* genus, in *Cryptococcus neoformans*, Mig1-mediated tolerance has been shown to limit treatment efficacy in murine cryptococcal meningitis through modulation of ergosterol synthesis (21). Despite these advances, the molecular mechanisms governing azole tolerance in *C. albicans* remain largely unknown.

Peroxisomes are single-membrane-bound organelles that coordinate essential metabolic processes in eukaryotes, including fatty acid β-oxidation, reactive oxygen species detoxification, and lipid homeostasis (22). Their biogenesis relies on a conserved set of peroxins (Pex proteins), which facilitate matrix protein import and membrane assembly (22). In pathogenic fungi, several peroxins such as Pex3, Pex5, and Pex10 have been linked to virulence through roles in cell wall integrity, morphogenesis, and adaptation to host-derived oxidative stress (23, 24). However, the function of the scaffolding peroxin Pex8 remains completely uncharacterized in human–pathogenic fungi *Candida* species. In particular, its potential involvement in antifungal drug responses and virulence in *C. albicans* has yet to be explored.

Given the critical gap in understanding the mechanisms of azole tolerance in *C. albicans*, and the unexplored role of peroxisomal components in fungal pathogenesis and drug response, this study aimed to investigate the potential function of the peroxisomal protein Pex8. We hypothesized that Pex8, as a core component of the peroxisomal import machinery, could play a previously unrecognized role in regulating cellular stress adaptation, thereby influencing antifungal drug susceptibility and virulence. Through a combination of genetic, transcriptomic, and lipidomic approaches, we sought to delineate the function of Pex8, providing new insights into the mechanisms of antifungal tolerance in this major human pathogen.

## RESULTS

### Pex8 modulates tolerance to azoles and amphotericin B

**Fig 1:**
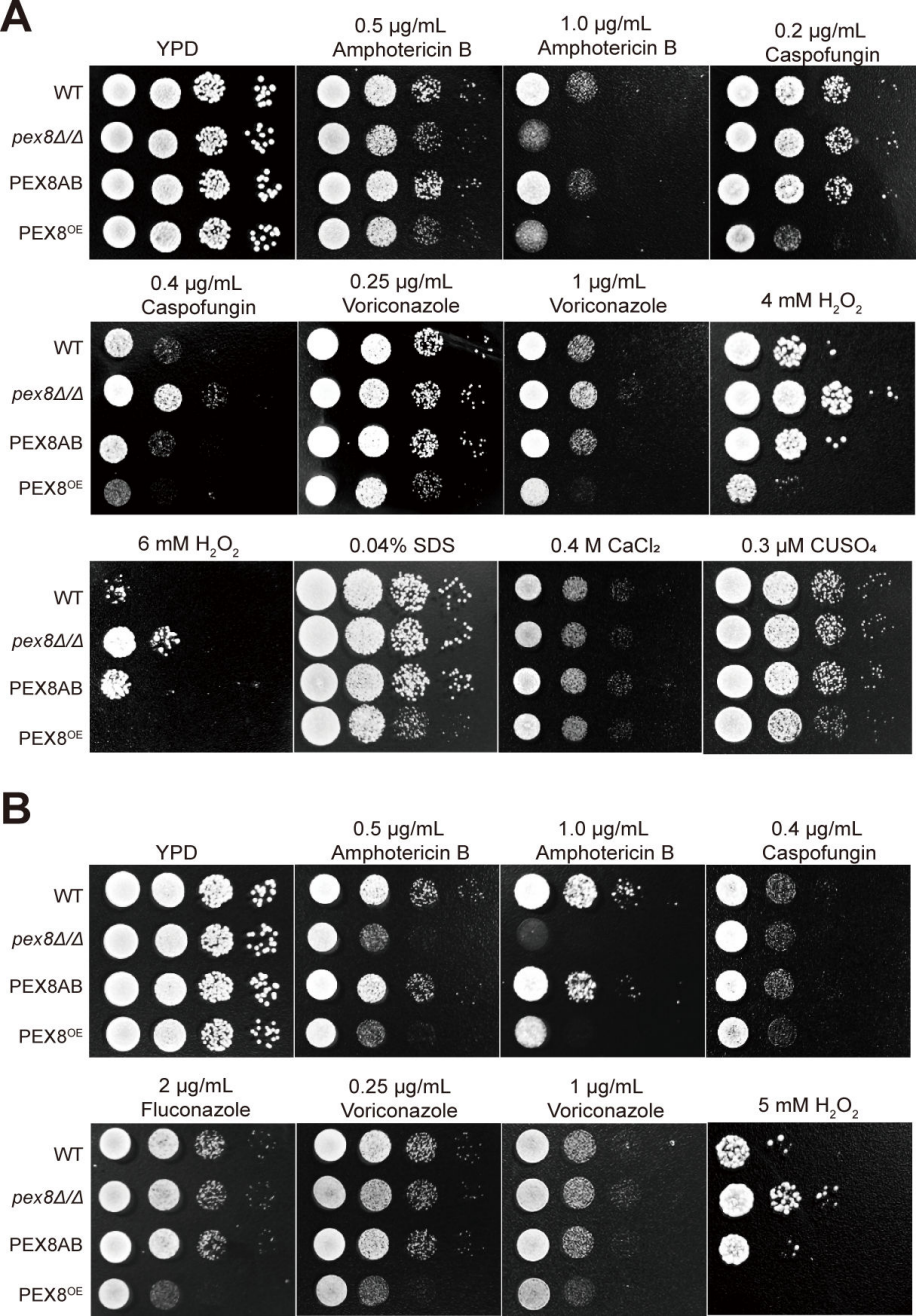
Phenotypic analysis of *PEX8* genetic modifications under different stress conditions at 30°C and 37°C. (**A**) Spot assay of *PEX8* knockout (*pex8Δ/Δ*) and overexpressing (*PEX8*^OE^) strains at 30°C. Wild-type (WT), *pex8Δ/Δ*, *PEX8*^OE^, and complemented (*PEX8AB*) strains were cultured overnight, washed with PBS, and then adjusted to 10^7^ cells/mL. Ten-fold serial dilutions (5 μL) of these strains were spotted on YPD plates containing the indicated stressors (H_2_O_2_, amphotericin B, caspofungin, voriconazole, CuSO_4_, CaCl_2_, or SDS) and incubated at 30°C for 24 h. (**B**) Spot assay of *PEX8* genetic modifications at host-relevant 37°C. Ten-fold serial dilutions of these strains were spotted on YPD plates containing the indicated stressors (H_2_O_2_, amphotericin B, caspofungin, voriconazole, or fluconazole) and incubated at 37°C for 24 h.

We identified the peroxisomal gene *PEX8* in *C. albicans* as a factor influencing stress responses and antifungal drug susceptibility in a temperature-dependent manner. Initial phenotypic screening using spot assays at 30°C revealed that *PEX8* deletion (*pex8Δ/Δ*) conferred enhanced growth in the presence of hydrogen peroxide, increased sensitivity to amphotericin B (AmB), and enhanced growth in the presence of caspofungin, without altering responses to copper ions, calcium ions, or SDS stress (Fig. 1A). Conversely, *PEX8* overexpression (*PEX8*^OE^) resulted in hypersensitivity to multiple stressors, including hydrogen peroxide, AmB, caspofungin, voriconazole, copper ions, and SDS. At the physiologically relevant temperature of 37°C, *pex8Δ/Δ* retained heightened sensitivity to amphotericin B and enhanced growth under hydrogen peroxide exposure, yet displayed susceptibility profiles similar to the wild-type (WT) strain in response to caspofungin and voriconazole (Fig. 1B). The *PEX8*^OE^ strain exhibited marked hypersensitivity to AmB and azole drugs (fluconazole and voriconazole) as well as hydrogen peroxide while maintaining normal caspofungin sensitivity. These temperature-dependent phenotypic shifts demonstrate that Pex8 modulates stress adaptation and antifungal drug responses in an environmentally conditioned manner. Given the physiological relevance of 37°C for this human pathogen, subsequent mechanistic studies focused on this temperature to elucidate the role of Pex8 in antifungal drug tolerance regulation.

**Fig 2:**
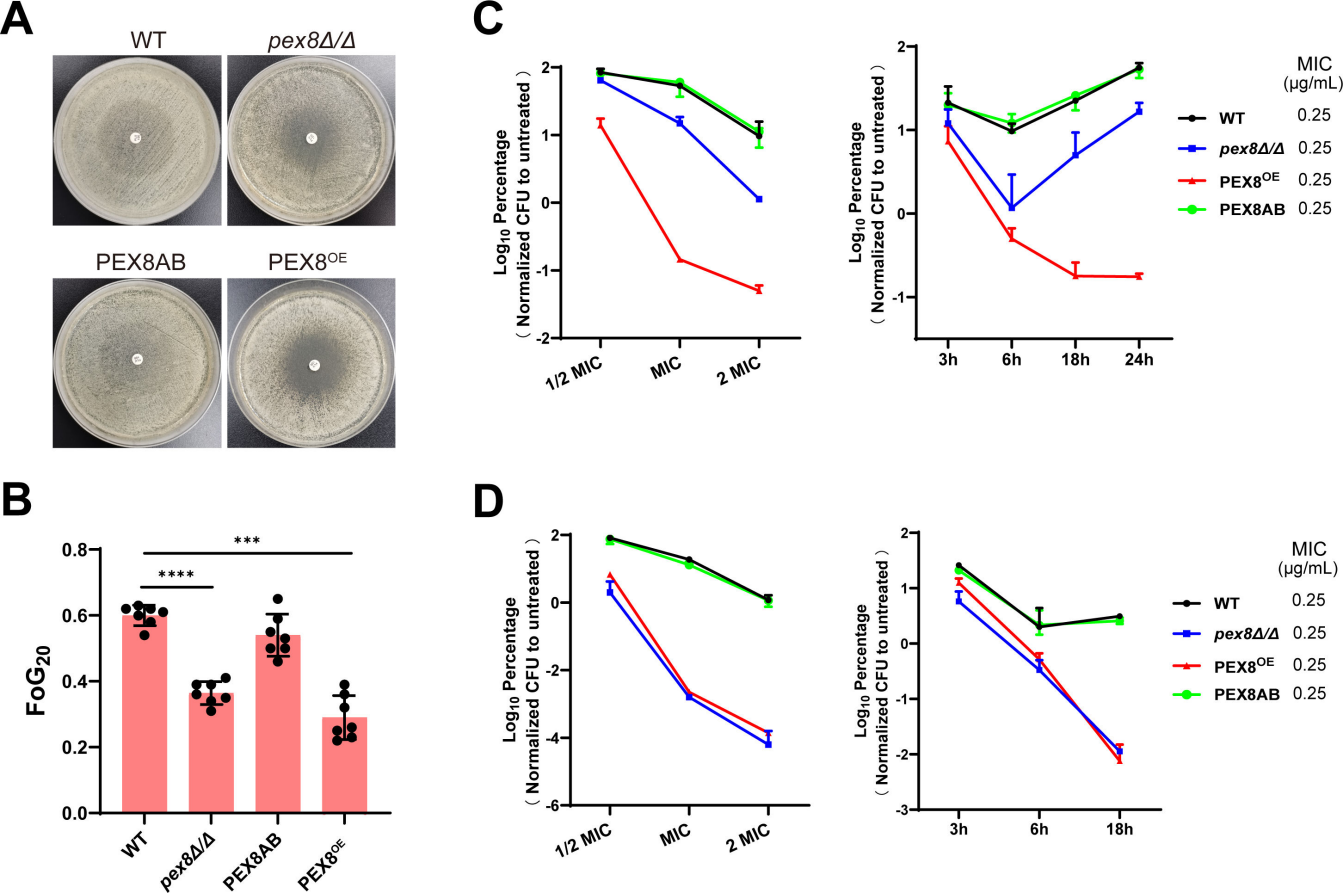
Pex8 modulates antifungal drug tolerance without altering resistance phenotypes. (**A**) Disk diffusion assays for fluconazole. Wild-type (WT), *pex8Δ/Δ*, *PEX8*^OE^, and complemented (*PEX8AB*) strains were grown on YPD agar for 24 h. Colonies were resuspended in sterile 0.9% saline and adjusted to 10^7^ cells/mL, and 100 μL of each suspension was spread onto fresh YPD agar. A disk containing 100 μg fluconazole was applied to each plate. Plates were incubated at 37°C for 24 h and photographed. (**B**) Quantitative analysis of fluconazole tolerance. Images from panel A were analyzed using diskImageR to calculate the fraction of growth within the inhibition zone (FoG_20_). Data represent the mean ± SD of seven biological replicates. Significance was determined by one-way ANOVA with Dunnett’s test (***P < 0.001, ****P < 0.0001). (**C**) Time–kill and dose–response analysis for fluconazole. (Left) Dose-dependent survival after 24 h: overnight cultures of WT, *pex8Δ/Δ*, *PEX8*^OE^, and *PEX8AB* strains were adjusted to 10^6^ cells/mL in PBS and treated with 0.5×, 1×, or 2× MIC fluconazole in YPD medium at 37°C. Survival rates were calculated by comparing CFU counts on YPD plates to untreated controls (set as 100%). (Right) Time–course survival at 1× MIC: Cells were harvested at 3, 6, 18, and 24 h. (**D**) Time–kill and dose–response analysis for amphotericin B (AmB). (Left) Dose-dependent survival after 24 h: strains were prepared as in panel C and treated with 0.5×, 1×, or 2× MIC AmB. Survival was normalized to untreated controls. (Right) Time–course survival at 1× MIC: cells were harvested at 3, 6, and 18 h. For all survival assays, viability was determined by CFU counting on YPD agar. Data are presented as the mean ± SD of three biological replicates.

To distinguish between resistance and tolerance phenotypes, we first determined MICs using broth microdilution assays. Both *pex8Δ/Δ* and *PEX8*^OE^ strains showed MICs identical to the WT strain for fluconazole (0.12 μg/mL) and voriconazole (0.015 μg/mL), indicating no change in classical resistance. To assess drug tolerance, we first employed disk diffusion assays, which revealed visibly reduced growth within the inhibition zones for both *pex8Δ/Δ* and *PEX8*^OE^ strains compared to WT and complemented strains (Fig. 2A). Quantitative analysis using the diskImageR pipeline was then performed to enable precise measurement of fluconazole tolerance, expressed as the fraction of growth within the inhibition zone (FoG_20_). The FoG_20_ values were significantly lower in both *pex8Δ/Δ* and *PEX8*^OE^ strains compared to the WT and complemented controls (Fig. 2B), confirming a substantial decrease in azole tolerance resulting from *PEX8* perturbation. We note that visual inspection of the plates indicated very faint residual growth within the inhibition zone of the *pex8Δ/Δ* strain that fell below the 20% threshold used for FoG_20_ calculation; thus, the metric may not capture extremely low levels of intra-halo growth. Finally, time–kill and dose–response assays under fluconazole exposure further substantiated the loss of tolerance, particularly in the *PEX8*^OE^ strain, where the fungistatic drug exhibited fungicidal activity (Fig. 2C).

For AmB, all strains showed equivalent MICs (0.25 μg/mL). Strikingly, survival assays under AmB pressure demonstrated dramatically enhanced killing efficiency in both *pex8Δ/Δ* and *PEX8*^OE^ strains. At 1× MIC after 18 h incubation, viability decreased by >100-fold compared to WT and complemented strains (Fig. 2D). This hypersensitivity occurred across multiple time points and drug concentrations, indicating Pex8 plays a crucial role in AmB tolerance regardless of expression level alteration.

### Pex8 overexpression downregulates ergosterol biosynthesis genes, potentially contributing to reduced fluconazole tolerance

**Fig 3:**
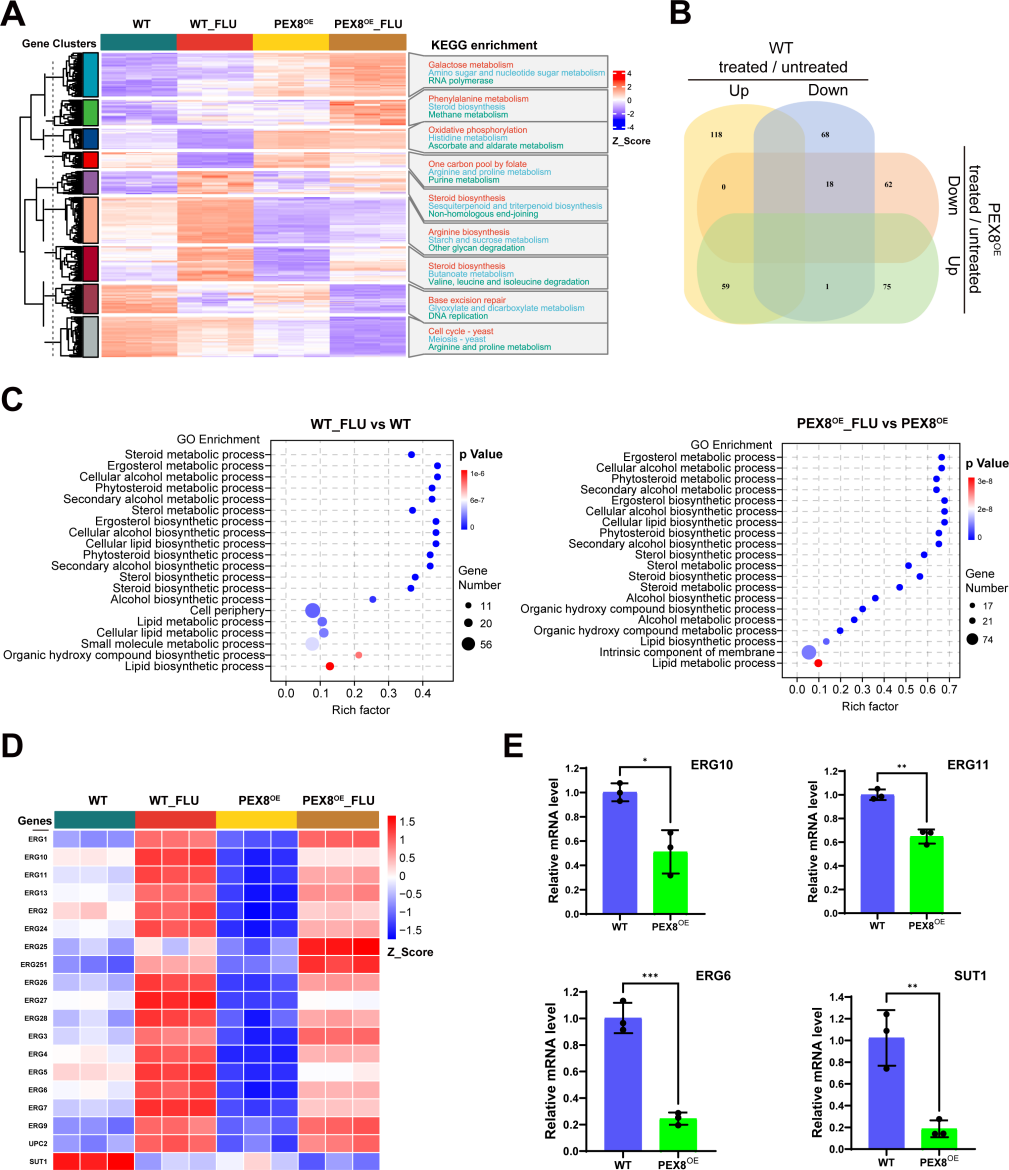
Pex8 overexpression attenuates fluconazole tolerance through transcriptional repression of ergosterol biosynthesis pathway. (**A**) Transcriptomic heatmap and KEGG enrichment reveal global transcriptional reprogramming. WT_FLU and *PEX8*^OE^_FLU denote WT and *PEX8*^OE^ strains treated with 2 µg/mL fluconazole for 30 min. Each condition includes three biological replicates. (**B**) Venn diagram illustrates the overlap between differentially expressed genes (DEGs) identified in WT and *PEX8*^OE^ strains following fluconazole treatment. (**C**) Gene Ontology enrichment of fluconazole responsive genes. Bubble plots display the top 20 significantly enriched GO terms for DEGs in WT (left panel) and *PEX8*^OE^ (right panel) strains. (**D**) Heatmap shows expression levels of ergosterol biosynthesis-associated DEGs across all experimental groups. (**E**) RT-qPCR validation of key ergosterol-related genes. Relative mRNA levels of *SUT1*, *ERG11*, *ERG6*, and *ERG10* normalized to the *ACT1* reference gene. Data represent the mean ± SD of three biological replicates. Statistical significance was determined by unpaired two-tailed Student’s *t*-test (*P < 0.05, **P < 0.01, ***P < 0.001 vs WT control).

Prompted by the consistent loss of azole tolerance in *PEX8*^OE^ strains, we employed RNA-seq to explore the underlying transcriptional changes. RNA-seq revealed distinct differential gene expression patterns between WT and *PEX8*^OE^ strains following fluconazole treatment (Fig. 3A and B). The WT strain exhibited 264 differentially expressed genes (DEGs; 177 upregulated, 87 downregulated), while the *PEX8*^OE^ strain showed 215 DEGs (135 upregulated, 80 downregulated) (Table S1). Notably, hierarchical clustering identified two significant gene clusters enriched in sterol biosynthetic pathways in both strains (Fig. 3A). Gene Ontology (GO) enrichment analyses further revealed that DEGs from both strains were specifically overrepresented in “ergosterol biosynthetic process” and “ergosterol metabolic process” (Fig. 3C; Table S1). Focusing on ergosterol-related genes, we observed marked downregulation of a broad set of ergosterol biosynthetic genes in the *PEX8*^OE^ strain compared to the wild type (Fig. 3D). RT-qPCR independently validated the reduced expression of key ergosterol biosynthesis genes (*ERG6*, *ERG10*, *ERG11*, and *SUT1*) in *PEX8*^OE^ strains (Fig. 3E). These transcriptional changes suggest a link between Pex8 overexpression and altered ergosterol pathway activity. However, future studies directly quantifying cellular sterol content will be required to confirm whether the observed transcriptional downregulation translates to reduced ergosterol biosynthesis. Together, these results indicate that Pex8 may influence azole tolerance, at least in part, through transcriptional modulation of ergosterol biosynthesis genes.

### Pex8 orchestrates AmB tolerance through comprehensive membrane lipid reprogramming

**Fig 4:**
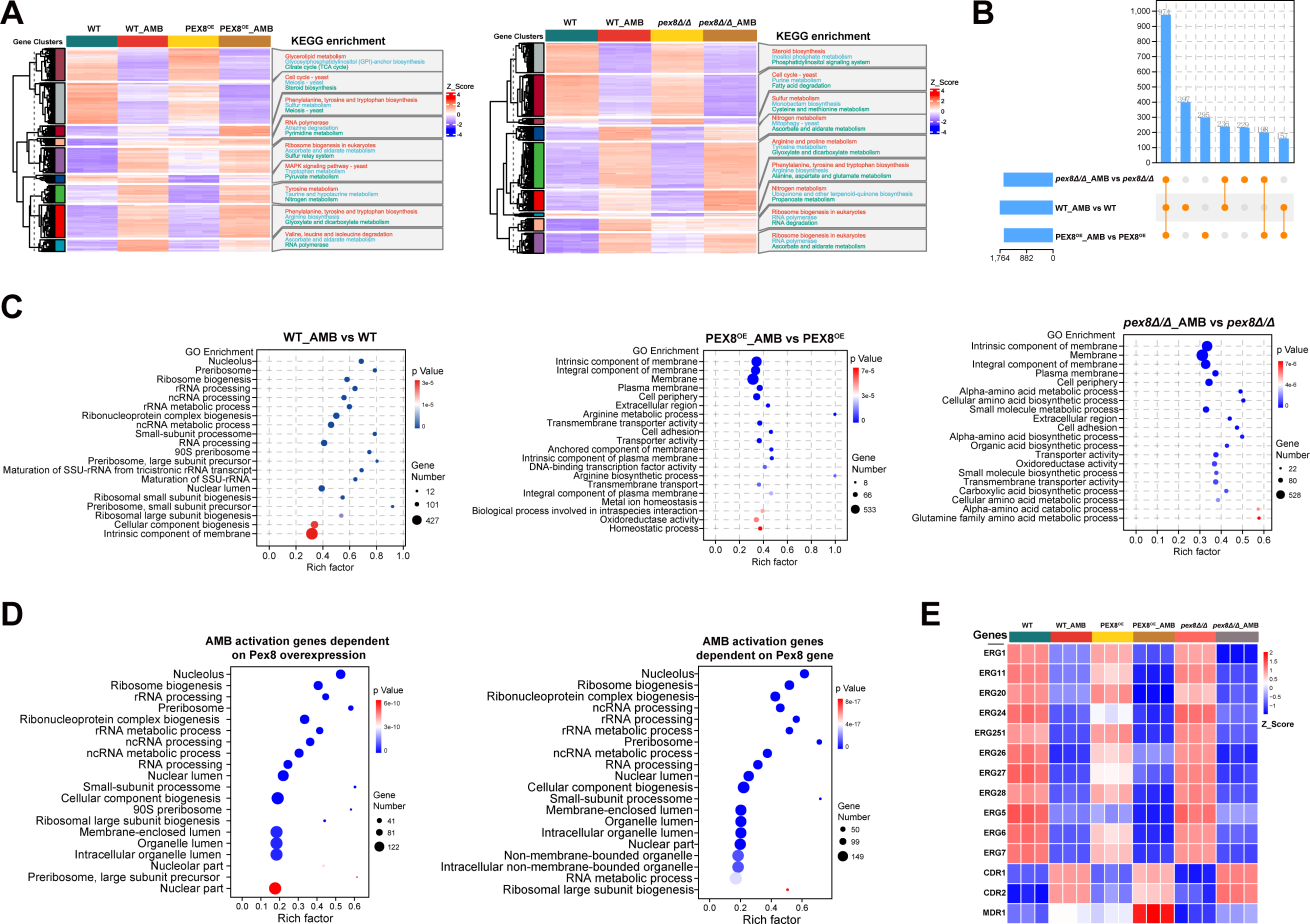
Comparative transcriptomic analysis reveals Pex8 modulates amphotericin B tolerance through membrane homeostasis regulation. (**A**) Transcriptomic heatmap and KEGG enrichment reveal global transcriptional reprogramming. WT_AMB, *PEX8*^OE^_AMB, and *pex8Δ/Δ*_AMB denote WT, *PEX8*^OE^, and *pex8Δ/Δ* strains treated with 0.25 µg/mL amphotericin B for 30 min. Each condition comprises three biological replicates. (**B**) UpSet plot quantifies the overlap among differentially expressed genes (DEGs) induced by amphotericin B in WT, *PEX8*^OE^, and *pex8Δ/Δ* strains. Vertical bars (left axis) indicate the number of DEGs belonging to each intersection set, labeled below with the corresponding combination of groups. The horizontal matrix (bottom) shows group membership for each set: filled orange dots denote which groups contribute to the intersection. For example, the tallest bar (leftmost) represents the 974 DEGs common to all three groups, whereas subsequent bars depict DEGs unique to individual group or shared between two groups. (**C**) Gene Ontology enrichment of amphotericin B-responsive genes. Bubble plots display the top 20 significantly enriched GO terms for DEGs in WT (left), *PEX8*^OE^ (middle), and *pex8Δ/Δ* strains (right). (**D**) Bubble plots display the top 20 significantly enriched GO terms for AmB-activated DEGs that specifically depend on Pex8 overexpression (left) or on Pex8 presence (right). (**E**) Heatmap shows expression profiles of ergosterol biosynthesis-associated DEGs across all experimental groups.

To elucidate the mechanistic basis of Pex8-mediated AmB tolerance, we conducted genome-wide transcriptional profiling of WT, *pex8Δ/Δ*, and *PEX8*^OE^ strains following AmB exposure. RNA-seq analysis identified substantial transcriptional reprogramming across all genotypes, with 1,764 DEGs (1,074 upregulated, 690 downregulated) in WT; 1,624 DEGs (998 upregulated, 626 downregulated) in *PEX8*^OE^; and 1,637 DEGs (957 upregulated, 680 downregulated) in *pex8Δ/Δ* (Fig. 4A and B; Table S1). Notably, 974 conserved DEGs (representing 55%–60% of strain-specific responses) formed a core AmB-responsive regulon, prominently enriched in ribosome biogenesis and steroid biosynthesis pathways (Fig. 4A and B). GO analysis further uncovered genotype-specific signatures: in the wild type, AmB-responsive DEGs were enriched for ribosome biogenesis and RNA metabolism; in *PEX8*^OE^ and *pex8Δ/Δ*, they were enriched for plasma membrane components (Fig. 4C; Table S1). DEGs dependent on Pex8 overexpression were additionally enriched in RNA processing, whereas AmB-activated genes dependent on Pex8 were enriched in both RNA processing and ribosome biogenesis (Fig. 4D). Notably, all strains exhibited significant downregulation of ergosterol biosynthesis genes following AmB treatment (Fig. 4E), despite their differential AmB tolerance, suggesting non-ergosterol membrane components may play a pivotal role in Pex8-mediated AmB tolerance.

**Fig 5:**
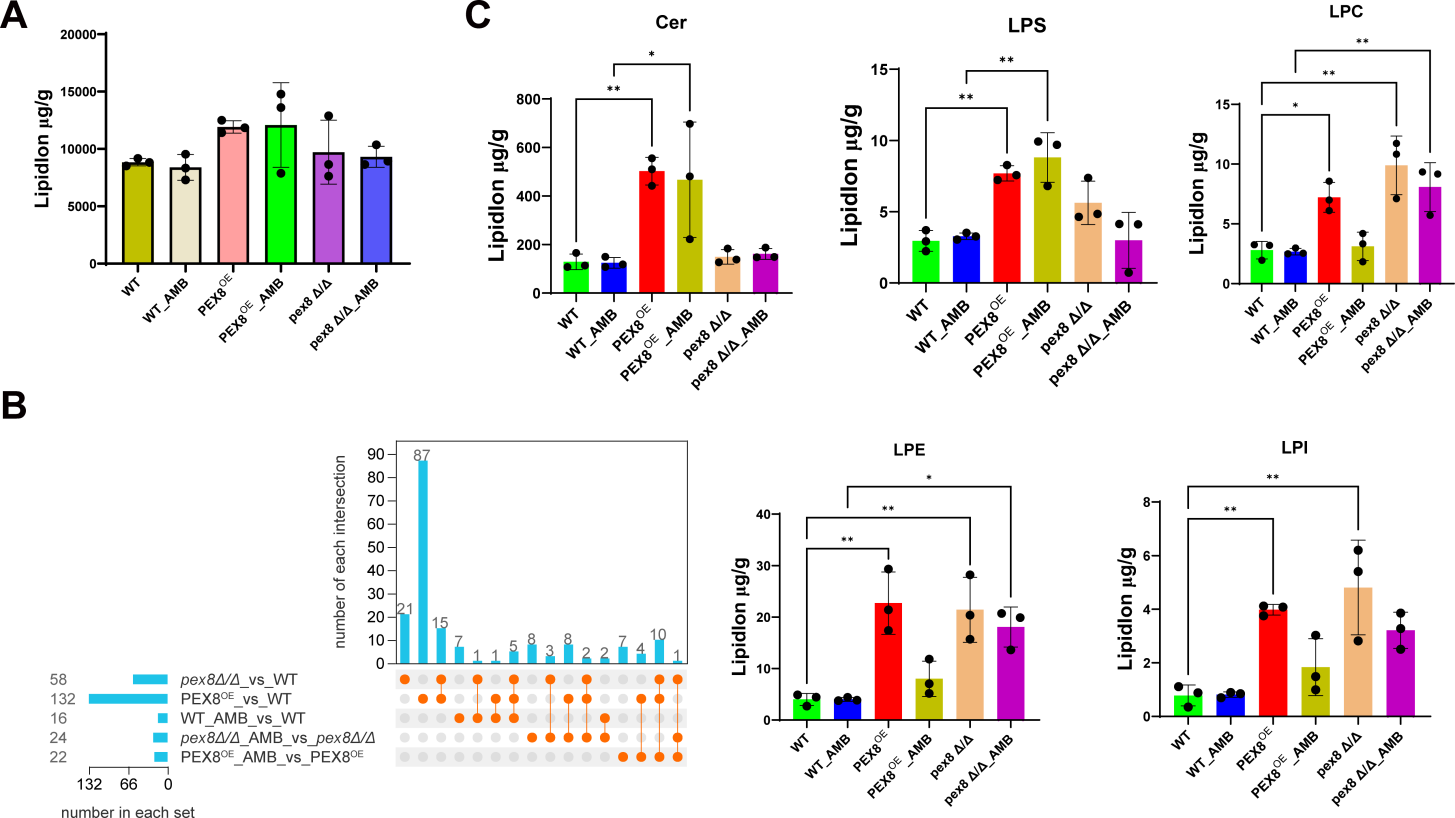
Pex8 orchestrates amphotericin B tolerance through comprehensive membrane lipid reprogramming. (**A**) Total lipid content remained unchanged across all strains. (**B**) UpSet plot displays overlaps of significantly changed lipid species across experimental groups. Vertical bars (left axis) indicate the number of differentially abundant lipids belonging to each intersection set, with the corresponding group combination labeled below. The horizontal matrix (bottom) illustrates set membership: filled orange dots denote which groups contribute to each intersection. For example, the leftmost vertical bar represents the 21 lipid species uniquely altered in the *pex8Δ/Δ* vs WT comparison, while the horizontal bar for the *pex8Δ/Δ* vs WT set shows that this group contains a total of 58 significantly changed lipids. Subsequent bars depict lipids unique to other groups or shared between two or three groups. (**C**) Expression changes of selected lipid subclasses. Significance was determined by one-way ANOVA with Tukey’s post hoc test (*P < 0.05, **P < 0.01).

To test whether membrane lipids other than ergosterol contribute to Pex8-mediated AmB tolerance, we performed untargeted lipidomic profiling across all strains. Total lipid abundance remained unchanged across all strains and conditions (Fig. 5A). Unsupervised clustering of differential lipid species, however, revealed that *PEX8*^OE^ and *pex8Δ/Δ* exhibited markedly divergent trajectories compared with WT upon AmB challenge (Fig. S1). In the absence of AmB, both *PEX8*^OE^ and *pex8Δ/Δ* strains underwent extensive lipid remodeling relative to the wild type, with 132 lipid species showing significant alterations in the *PEX8*^OE^ strain (Fig. 5B; Table S2). By contrast, AmB treatment itself induced only minor lipidomic shifts across all strains, as evidenced by merely 16 significantly changed lipid species in the wild-type strain following drug exposure (Fig. 5B; Table S2). Subclass analysis identified ceramides and lysophospholipids lysophosphatidylserine, lysophosphatidylethanolamine (LPE), and lysophosphatidylinositol (LPI) as significantly elevated in the *PEX8*^OE^ strain. In contrast, the *pex8Δ/Δ* strain showed selective accumulation of lysophosphatidylcholine, LPE, and LPI (Fig. 5C). This coordinated yet distinct remodeling—occurring despite conserved downregulation of ergosterol biosynthesis genes—suggests Pex8 orchestrates AmB tolerance through selective regulation of non-ergosterol membrane components, likely modulating drug membrane interactions via lipid asymmetry alterations.

### Pex8 overexpression impairs hyphal development and attenuates virulence in *C. albicans*

**Fig 6:**
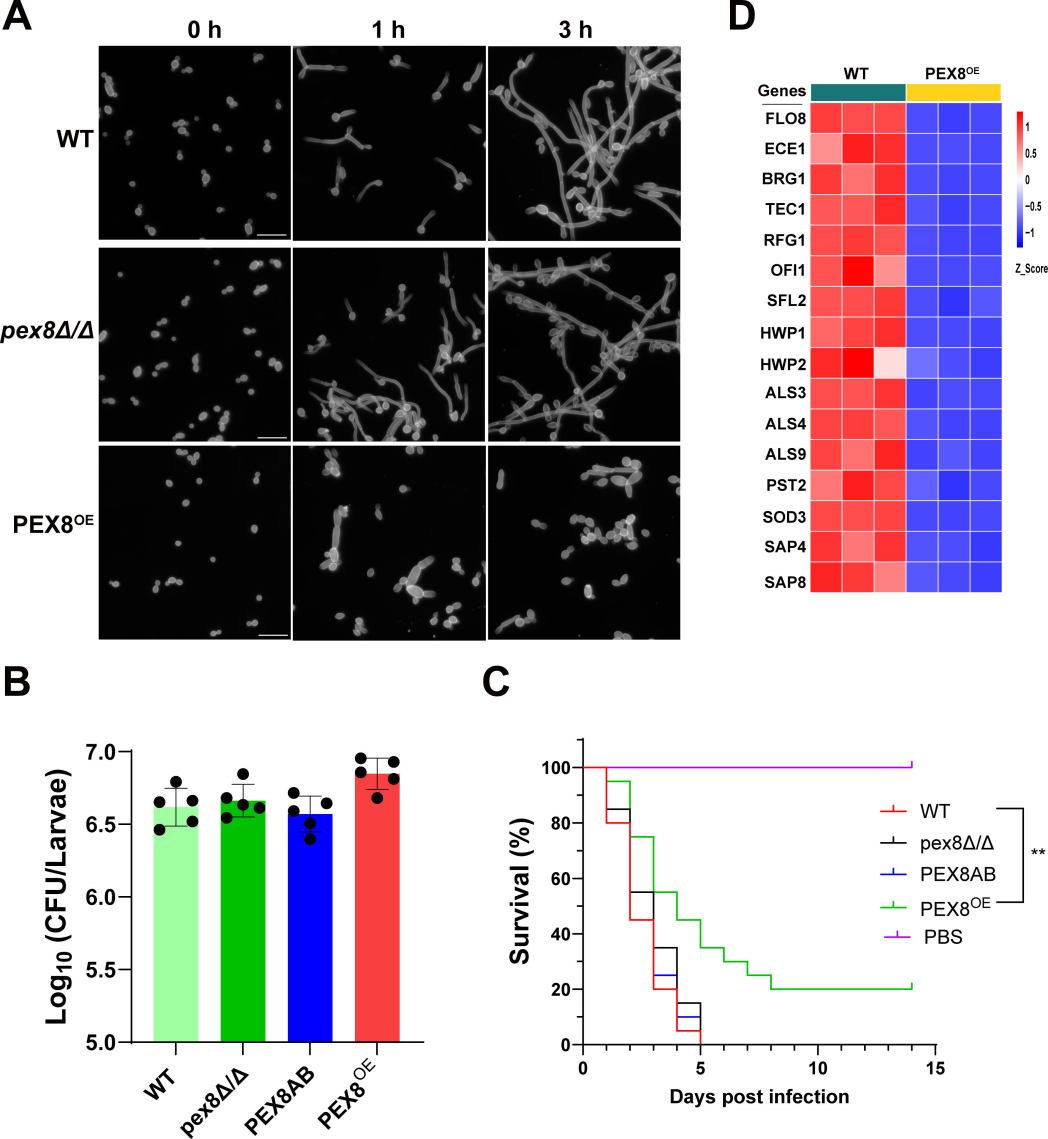
Pex8 governs filamentation and virulence. (**A**) Serum-induced hyphal formation. Strains were grown to logarithmic phase, washed twice with PBS, and adjusted to an optical density of 1.0 at 600 nm. Hyphal induction was performed in YPD medium supplemented with 10% fetal bovine serum at 37°C with shaking. Samples were collected at 0, 1, and 3 h post-induction, washed by centrifugation, and stained with calcofluor white for fluorescence microscopy imaging. Scale bar, 20 μm. (**B**) Fungal burden assessment in the *Galleria mellonella* infection model. Larvae infected with respective strains were homogenized, and tissue lysates were serially diluted. Aliquots (100 μL) were plated on YPD agar containing antibiotics. After 24 h of incubation at 37°C, colony-forming units (CFUs) were enumerated and normalized to CFU per larva. (**C**) Kaplan–Meier survival curves. Groups of larvae (*n* = 20 per group) were inoculated with designated strains. Survival was monitored over time, and Kaplan–Meier curves were generated. Statistical significance between groups was determined by log-rank (Mantel–Cox) test. (**D**) Heatmap illustrating RNAseq-derived expression changes of hyphal regulatory and virulence-associated genes in the *PEX8*^OE^ strain vs WT strain.

To investigate the role of Pex8 in virulence regulation, we first assessed hyphal formation under serum induction. Strikingly, the *PEX8*^OE^ strain exhibited severely impaired hyphal development, failing to form true hyphae, whereas WT and *pex8Δ/Δ* strains showed normal hyphal morphogenesis (Fig. 6A). In *Galleria mellonella* infection models, all strains established comparable tissue fungal burdens as measured by colony-forming unit (CFU) counts (Fig. 6B). It should be noted that CFU enumeration may systematically underestimate the total cellular load of filament-competent strains, as hyphal filaments can yield single colonies despite containing multiple cellular compartments. Nevertheless, the *PEX8*^OE^ strain exhibited significantly attenuated virulence, with 20% larval survival vs complete lethality (0% survival) for both the WT and *pex8Δ/Δ* strains within the same observation period (Fig. 6C). This marked attenuation in lethality indicates that *PEX8* overexpression specifically impairs the pathogenic capacity of *C. albicans*. RNA-seq profiling revealed that Pex8 overexpression markedly represses key hyphal regulatory genes (such as *FLO8*, *BRG1*, and *TEC1*) and concomitantly downregulates a suite of virulence-associated genes, including *ECE1*, ALS family members, and SAP proteases (Fig. 6D). These data indicate that Pex8 upregulation suppresses both hyphal growth pathways and virulence gene expression networks.

## DISCUSSION

In this study, we establish the peroxisomal protein Pex8 as a global regulator that coordinately controls antifungal tolerance, oxidative stress adaptation, and virulence in *C. albicans*. Our integrated analyses reveal that both deletion and overexpression of *PEX8* significantly reduce tolerance to azoles and amphotericin B without altering conventional MIC values. Transcriptomic profiling of *PEX8*-overexpressing strains demonstrated suppression of ergosterol biosynthesis genes, while lipidomic analysis of both knockout and overexpressing strains revealed extensive membrane lipid remodeling characterized by specific alterations in ceramide and lysophospholipid subclasses. These findings suggest distinct yet complementary mechanisms: azole tolerance attenuation through ergosterol pathway regulation and amphotericin B tolerance reduction through ergosterol-independent membrane reorganization. Phenotypically, *PEX8*-overexpressing cells are impaired in hyphal morphogenesis and exhibit attenuated virulence *in vivo*, whereas the *pex8Δ/Δ* mutant retains full pathogenicity, indicating that Pex8 acts as a negative regulator of filamentation and host lethality.

Notably, Pex8-dependent regulation of caspofungin tolerance exhibits marked temperature sensitivity. At 30°C, Pex8 deletion significantly enhanced tolerance to caspofungin, while its overexpression substantially compromised it, indicating that Pex8 acts as a negative regulator of caspofungin tolerance under this condition. However, this phenotype was absent at 37°C, where neither genetic perturbation affected caspofungin susceptibility. This temperature-dependent pattern aligns with previous observations that *C. albicans* displays significantly higher ketoconazole tolerance at 37°C than at 30°C (25). Conversely, *Cryptococcus neoformans* shows enhanced fluconazole susceptibility and diminished heteroresistance under elevated temperatures (26, 27). In contrast, Pex8 consistently negatively regulated oxidative stress responses regardless of temperature, with deletion conferring H_2_O_2_ tolerance and overexpression enhancing sensitivity. The observed divergence in temperature sensitivity between drug tolerance and stress response indicates distinct underlying regulatory mechanisms, underscoring the importance of employing host-relevant temperatures in antifungal research.

The observed reduction in azole tolerance following both deletion and overexpression of *PEX8* positions this peroxisomal factor as a potential target for mitigating tolerance-related treatment failures. This finding carries clinical importance, as conventional antifungal susceptibility testing fails to detect tolerant strains, which have been increasingly associated with persistent candidemia (17). A notable limitation in the existing literature is the frequent conflation of tolerance with resistance, often stemming from reliance on phenotypic screening methods such as spot assays and time–kill curves without accompanying MIC data (28–30). This methodological shortcoming, exacerbated by the historically ambiguous definition of antifungal tolerance, has hindered the precise functional annotation of putative tolerance regulators. Here, we clearly demonstrate that Pex8 overexpression diminishes azole tolerance without altering MIC, a phenotype that may be linked to its transcriptional suppression of ergosterol biosynthesis genes. This aligns with previous reports that transcriptional downregulation of ergosterol pathway genes reduces azole tolerance, whereas their upregulation enhances it (31, 32). While transcriptional profiling of the *pex8Δ/Δ* mutant under azole stress could further clarify core tolerance mechanisms, the overexpression strain provided a stronger phenotypic signal for initial mechanistic discovery. Collectively, our findings reveal a previously unrecognized functional connection between peroxisomal activity and ergosterol metabolism in the regulation of antifungal drug tolerance.

In addition, our study reveals that both genetic deletion and overexpression of *PEX8* result in reduced tolerance to AmB. Transcriptomic analysis indicated that AmB exposure downregulated ergosterol biosynthesis genes across all tested strains. Complementary lipidomic profiling revealed extensive membrane lipid remodeling characterized by specific alterations in ceramide and lysophospholipid subclasses, thereby indicating an ergosterol-independent mechanism of tolerance attenuation. These findings align with established literature: alterations in sterol biosynthesis are recognized contributors to AmB resistance in *Candida auris*, where mutations in *ERG3* have been identified as a key resistance mechanism in clinical isolates (33, 34). Similarly, modifications in sterol profiles have been associated with reduced AmB susceptibility in *Candida kefyr* (35). Given that AmB directly targets membrane ergosterol, our data suggest that Pex8-mediated lipid reorganization disrupts membrane architecture, thereby enhancing drug efficacy. As peroxisomes are conserved organelles integral to cellular lipid metabolism (36) and sterol homeostasis is critical for AmB activity, the interplay between peroxisomal function and membrane sterol dynamics represents a promising avenue for future research. Elucidating how peroxisomal proteins such as Pex8 coordinate lipid metabolic pathways may uncover novel mechanisms of AmB tolerance and inform strategies to overcome drug resistance.

A consideration in interpreting our findings is that the observed phenotypes may reflect a broader disruption of peroxisomal functions rather than a specific regulatory role of Pex8 itself. As versatile organelles, peroxisomes mediate cellular energy homeostasis, redox balance, and stress adaptation (37). In filamentous fungi, the peroxins have been shown to critically regulate hyphal growth and virulence; for instance, deletion of *AoPEX14/17* in *Arthrobotrys oligospora* severely impairs mycelial development, conidiation, trap formation, and pathogenicity (38–40). Although analogous functions have not yet been characterized in *Candida* species, our transcriptomic data reveal that *PEX8* overexpression downregulates key hyphal-specific genes (e.g., *FLO8*, *BRG1*, and *TEC1*) without markedly altering the expression of central regulators *EFG1* or *CPH1*. This pattern suggests that peroxisomal activity influences filamentation through a pathway that is largely parallel to, or converges downstream of, the canonical cAMP-PKA and MAPK cascades (41). Notably, although *PEX8* deletion and overexpression exerted opposing effects on H_2_O_2_ sensitivity, both perturbations similarly reduced tolerance to amphotericin B and azoles. This divergence implies that the role of Pex8 within the peroxisome may be distinct from its broader influence on antifungal drug susceptibility, a possibility that warrants further investigation. Our study thus provides the first evidence linking peroxisomal function, through Pex8, to antifungal drug tolerance in *C. albicans* while also highlighting the need to dissect whether this reflects a specific regulatory function of Pex8 or a more general consequence of perturbing peroxisomal homeostasis.

In summary, our study establishes Pex8 as a central regulator at antifungal tolerance, stress adaptation, and virulence in *C. albicans*. These findings not only expand our understanding of peroxisomal functions in fungal pathogenesis but also reveal Pex8-mediated pathways as potential targets for innovative antifungal strategies aimed at overcoming drug tolerance.

## MATERIALS AND METHODS

### Strains, media, and chemicals

All strains and plasmids used in this study are listed in Table S3. The *PEX8* knockout mutant (*pex8Δ/Δ*) and its complemented strain were described in our previous work (42). In this study, the *PEX8*-overexpressing strain was generated via homologous recombination. The native promoter of *PEX8* was replaced by the strong, constitutive *TDH3* promoter while retaining the endogenous coding sequence. A cassette containing the *SAT1* selectable marker flanked by the *TDH3* promoter was amplified and assembled with upstream and downstream homologous regions (flanking the *PEX8* start codon) into a pUC19 backbone (primers listed in Table S4). The resulting plasmid was linearized with KpnI and transformed into the wild-type strain using standard lithium acetate transformation. Transformants were selected on YPD plates containing 200 µg/mL nourseothricin. To verify correct genomic integration, positive clones were screened by PCR using primer pairs specific to the 5′ and 3′ homology junctions (Table S4). To ensure selection of a representative strain, we performed RT-qPCR to quantify *PEX8* expression and selected the clone with the highest expression level for subsequent experiments. To confirm that the observed phenotypes were due to *PEX8* overexpression and not to off-target integration events, three independent, PCR-verified *PEX8*-overexpressing transformants were subjected to key phenotypic assays, including azole and amphotericin B susceptibility tests and hyphal growth assessment. All three transformants showed consistent phenotypes (see Fig. S2), supporting the conclusion that the observed effects are directly linked to increased *PEX8* expression.

For long-term storage, strains were kept at −80°C in YPD medium (1% yeast extract, 2% peptone, and 2% glucose) containing 20% (vol/vol) glycerol. Routine cultures were grown in YPD at 37°C with shaking at 220 rpm. For antifungal susceptibility testing, strains were cultured in RPMI 1640 medium (with L-glutamine, without sodium bicarbonate; Sigma-Aldrich). Fluconazole, voriconazole, caspofungin, and amphotericin B were purchased from Selleck Chemicals.

### Spot assay

Strains were grown in YPD medium overnight, harvested by centrifugation, washed twice with sterile PBS, and suspended at 10⁷ cells/mL. Serial 10-fold dilutions were prepared, and 5 µL aliquots of each dilution were spotted onto YPD agar plates containing the indicated compounds. Plates were incubated at 30°C or 37°C for 24 h and then photographed. Initial phenotypic screening was performed at 30°C to broadly survey stress-response profiles, followed by validation at the host-relevant temperature of 37°C. The stressors were selected to probe functions linked to peroxisomal activity (H_2_O_2_), antifungal drug susceptibility, membrane integrity (SDS), and ion homeostasis (Cu²^+^ and Ca²^+^).

### Disk diffusion assay

The disk diffusion assays were performed following previously established methods with minor modifications (17). Briefly, tested strains were cultured on YPD agar plates for 24 h. Colonies were resuspended in sterile 0.9% saline, and the cell density was adjusted to 10^7^ cells/mL. One hundred microliters of the standardized suspension was spread onto fresh YPD agar plates (9 cm diameter). A fluconazole-impregnated disk (100 µg per disk; Liofilchem, Abruzzi, Italy) was aseptically placed at the center of each inoculated plate. Plates were incubated at 37°C for 24 h and photographed. Images were analyzed using the diskImageR pipeline (43) to quantify two key parameters: the fractional growth within the inhibition zone (FoG) and the zone radius (RAD).

### Survival assay

Yeast cells were cultured overnight in YPD at 37°C with 220 rpm shaking, washed twice in sterile PBS, and resuspended to 10⁶ cells/mL. Aliquots were transferred to fresh medium containing the stressor (drug-free medium served as negative control) and incubated at 37°C with shaking. At specified intervals, samples were removed and serially diluted in PBS, and 100 µL spots were plated on YPD agar. After 48 h incubation at 37°C, colonies were counted and survival expressed as (CFU_treatment / CFU_control) × 100%. Experiments were performed in triplicate with independent cultures.

### RNA sequencing and bioinformatics analysis

The strains WT, *pex8Δ/Δ*, and *PEX8*^OE^ were precultured in YPD medium at 37°C with shaking to mid-logarithmic phase. Cells were harvested by centrifugation, washed twice with sterile PBS, and resuspended in fresh YPD medium to an OD600 of 2.0. Cultures were then divided, and fluconazole (final concentration 2 µg/mL) or amphotericin B (final concentration 0.25 µg/mL) was added. The cultures were incubated with shaking at 37°C for exactly 30 min before rapid harvesting for RNA extraction. Subsequently, RNA-seq analysis was performed as previously described (42). In brief, total RNA was extracted using the Quick-RNA Microprep Kit (Zymo Research, Irvine, CA, USA). Sequencing libraries were prepared following the Illumina Stranded mRNA protocol and paired-end sequenced (2 × 150 bp) on the Illumina NovaSeq 6000 platform through Shanghai Personal Biotechnology Co., Ltd., achieving >20 million reads per sample. Clean reads were aligned to the *C. albicans* SC5314 reference genome (GenBank: GCF_000182965.3). Statistical analysis of gene expression was performed using DESeq2 (v1.34.0) in R (v4.1.2). Genes were considered differentially expressed if they exhibited a fold change of ≥1.5 and a *P* value of <0.05. Three independent biological replicates were analyzed for each condition. Functional enrichment analysis was performed using clusterProfiler (v4.2.2) with GO and KEGG pathway databases.

### Quantitative real-time PCR

Total RNA was extracted from log-phase yeast cultures using the Quick-RNA Microprep kit (Zymo Research, Irvine, CA, USA), followed by cDNA synthesis with PrimeScript RT reagent (TaKaRa, Tokyo, Japan). Gene expression analysis was performed using SYBR Premix Ex Taq II (Tli RNaseH Plus) with gene-specific primers (Table S4). Transcript abundance was calculated by the 2^–ΔΔCt^ method, with *ACT1* serving as the constitutive reference. Data from three independent biological replicates were used for statistical analysis.

### Lipidomics analysis

Lipidomic profiling was performed as previously described (44). Briefly, lipids were extracted from cell pellets using methyl tert-butyl ether/methanol, separated by UHPLC on a C18 column with a 25-min acetonitrile/isopropanol gradient, and analyzed by Q Exactive HF-X mass spectrometry in data-dependent acquisition mode. Lipid identification and quantification were conducted using LipidSearch v4.2, with differential lipids determined by OPLS-DA (VIP > 1) and Student’s *t*-test (*P* < 0.05).

### *Galleria mellonella* infection model

The experimental procedures were performed as previously described (20). Briefly, healthy *G. mellonella* larvae (Keyun Biotechnology Company, China) were randomly allocated into experimental groups (*n* = 20/group). Fungal infection was established by injecting 10 μL of *C. albicans* suspension (1×10^6 CFU) into the last left proleg. Control larvae received equal volumes of sterile PBS. For survival analysis, infected larvae were maintained at 37°C and monitored daily for 14 days. To quantify fungal burden, five larvae per group were homogenized at 24 h post-infection using a FastPrep-24 5G system (6.0 m/s, 40 sec). Serial dilutions of homogenates were plated on YPD agar, and CFUs were enumerated after 24 h incubation at 30°C.

## Data Availability

All data supporting this study are provided within the article and its supplementary materials. Raw RNA-seq files are publicly available in the NCBI Sequence Read Archive under BioProject PRJNA1345091. Untargeted lipidomics data sets have been deposited in the EMBL-EBI MetaboLights repository under accession number MTBLS13170.

## SUPPLEMENTAL MATERIAL

Fig. S1 aac.01662-25-s0001.tif Trend clustering analysis was employed to elucidate the expression patterns and dynamic changes of all identified lipid molecules across different groups.

Fig. S2 aac.01662-25-s0002.tif PEX8 overexpression reduces antifungal drug susceptibility and impairs hyphal morphogenesis.

Supplemental material aac.01662-25-s0003.docx Supplemental figure and table legends.

Table S1 aac.01662-25-s0004.xlsx Differentially expressed genes analysis.

Table S2 aac.01662-25-s0005.xlsx Differentially abundant lipid species.

Table S3 aac.01662-25-s0006.xlsx Strains and plasmids used in this study.

Table S4 aac.01662-25-s0007.xlsx Primers used in this study.

ASM does not own the copyrights to Supplemental Material that may be linked to, or accessed through, an article. The authors have granted ASM a non-exclusive, world-wide license to publish the Supplemental Material files. Please contact the corresponding author directly for reuse.

## References

[1] Giannella M, Lanternier F, Dellière S, Groll AH, Mueller NJ, Alastruey-Izquierdo A, Slavin MA, ECCMID study groups on Invasive Fungal Infection and Infection in Immunocompromised Hosts. 2025. Invasive fungal disease in the immunocompromised host: changing epidemiology, new antifungal therapies, and management challenges. Clin Microbiol Infect 31:29–36. doi:10.1016/j.cmi.2024.08.006 39142631

[2] Lass-Flörl C, Kanj SS, Govender NP, Thompson GR 3rd, Ostrosky-Zeichner L, Govrins MA. 2024. Invasive candidiasis. Nat Rev Dis Primers 10:20. doi:10.1038/s41572-024-00503-3 38514673

[3] Valentine M, Wilson D, Gresnigt MS, Hube B. 2025. Vaginal Candida albicans infections: host-pathogen-microbiome interactions. FEMS Microbiol Rev 49:fuaf013. doi:10.1093/femsre/fuaf013 40347186 PMC12071381

[4] Kriegl L, Egger M, Boyer J, Hoenigl M, Krause R. 2025. New treatment options for critically important WHO fungal priority pathogens. Clin Microbiol Infect 31:922–930. doi:10.1016/j.cmi.2024.03.006 38461942

[5] Vazquez JA, Whitaker L, Zubovskaia A. 2025. Invasive candidiasis in the intensive care unit: where are we now? J Fungi (Basel) 11:258. doi:10.3390/jof11040258 40278079 PMC12028288

[6] Schille TB, Sprague JL, Naglik JR, Brunke S, Hube B. 2025. Commensalism and pathogenesis of Candida albicans at the mucosal interface. Nat Rev Microbiol 23:525–540. doi:10.1038/s41579-025-01174-x 40247134

[7] Robbins N, Cowen LE. 2025. Candida. Curr Biol 35:R522–R526. doi:10.1016/j.cub.2025.01.063 40494308

[8] Lin J, Filler SG. 2025. Host targets of candidalysin. PLoS Pathog 21:e1013284. doi:10.1371/journal.ppat.1013284 40549807 PMC12184922

[9] Lindemann-Perez E, Perez JC. 2024. Candida albicans natural diversity: a resource to dissect fungal commensalism and pathogenesis. Curr Opin Microbiol 80:102493. doi:10.1016/j.mib.2024.102493 38833793 PMC12743322

[10] Ribeiro F de C, Kemmerich KK, Gonçale JC, Junqueira JC, Mannan M, Nabeela S, Colombo AL, Uppuluri P. 2025. Candida albicans recovered from persistent candidemia exhibits enhanced virulence traits. J Infect Dis 231:e803–e812. doi:10.1093/infdis/jiae631 39693248 PMC11998578

[11] Al Benwan K, Ahmed S, Al Banwan D, John M. 2025. Candidemia in a general hospital in Kuwait: epidemiology, species distribution, risk factors, and antifungal susceptibility patterns over a 10-year period (2015–2024). J Fungi (Basel) 11:670. doi:10.3390/jof11090670 41003216 PMC12471101

[12] Xiao M, Sun Z-Y, Kang M, Guo D-W, Liao K, Chen S-A, Kong F, Fan X, Cheng J-W, Hou X, Zhou M-L, Li Y, Yu S-Y, Huang J-J, Wang H, Xu Y-C, China Hospital Invasive Fungal Surveillance Net (CHIF-NET) Study Group. 2018. Five-year national surveillance of invasive candidiasis: species distribution and azole susceptibility from the China hospital invasive fungal surveillance net (CHIF-NET) study. J Clin Microbiol 56:e00577-18. doi:10.1128/JCM.00577-18 29743305 PMC6018329

[13] Li Y, Hind C, Furner-Pardoe J, Sutton JM, Rahman KM. 2025. Understanding the mechanisms of resistance to azole antifungals in Candida species. JAC Antimicrob Resist 7:dlaf106. doi:10.1093/jacamr/dlaf106 40583995 PMC12205959

[14] Bavaro DF, Diella L, De Angelis A, Vanoni M, Belati A, De Santis L, Bussini L, Montemurro L, Papale R, Ronga L, et al.. 2025. Why do echinocandins fail? Identifying key predictors to improve clinical outcomes of candida bloodstream infections: a retrospective multicenter cohort study. Int J Infect Dis 160:108046. doi:10.1016/j.ijid.2025.108046 40912531

[15] Tiseo G, Vena A, Bassetti M, Bartalucci C, Cerchiaro M, Cesaretti M, Marchese A, Di Pilato V, Escribano P, Forniti A, Giacobbe DR, Guinea J, Limongelli A, Lupetti A, Machado M, Mikulska M, Salmanton-García J, Soriano-Martin A, Taramasso L, Valerio M, Bouza E, Muñoz P, Falcone M, ESCMID Fungal Infection Study Group (EFISG). 2025. Persistent candidemia caused by different Candida species: data from a multicenter contemporary cohort. J Infect 91:106586. doi:10.1016/j.jinf.2025.106586 40816720

[16] Levinson T, Dahan A, Novikov A, Paran Y, Berman J, Ben-Ami R. 2021. Impact of tolerance to fluconazole on treatment response in Candida albicans bloodstream infection. Mycoses 64:78–85. doi:10.1111/myc.13191 33000505 PMC7614594

[17] Rosenberg A, Ene IV, Bibi M, Zakin S, Segal ES, Ziv N, Dahan AM, Colombo AL, Bennett RJ, Berman J. 2018. Antifungal tolerance is a subpopulation effect distinct from resistance and is associated with persistent candidemia. Nat Commun 9:2470. doi:10.1038/s41467-018-04926-x 29941885 PMC6018213

[18] Delarze E, Brandt L, Trachsel E, Patxot M, Pralong C, Maranzano F, Chauvel M, Legrand M, Znaidi S, Bougnoux M-E, d’Enfert C, Sanglard D. 2020. Identification and characterization of mediators of fluconazole tolerance in Candida albicans. Front Microbiol 11:591140. doi:10.3389/fmicb.2020.591140 33262748 PMC7686038

[19] Zhu Q, Wijnants S, Feil R, Van Genechten W, Vergauwen R, Van Goethem O, Lunn JE, Van Ende M, Van Dijck P. 2025. The stress-protectant molecule trehalose mediates fluconazole tolerance in Candida glabrata. Antimicrob Agents Chemother 69:e0134924. doi:10.1128/aac.01349-24 39853120 PMC11881567

[20] Wu Y, Zou Y, Dai Y, Lu H, Zhang W, Chang W, Wang Y, Nie Z, Wang Y, Jiang X. 2025. Adaptive morphological changes link to poor clinical outcomes by conferring echinocandin tolerance in Candida tropicalis. PLoS Pathog 21:e1013220. doi:10.1371/journal.ppat.1013220 40424325 PMC12140413

[21] Chen L, Tian X, Zhang L, Wang W, Hu P, Ma Z, Li Y, Li S, Shen Z, Fan X, Ye L, Ke W, Wu Y, Shui G, Xiao M, He G-J, Yang Y, Fang W, Bai F, Liao G, Chen M, Lin X, Li C, Wang L. 2024. Brain glucose induces tolerance of Cryptococcus neoformans to amphotericin B during meningitis. Nat Microbiol 9:346–358. doi:10.1038/s41564-023-01561-1 38225460

[22] Kumar R, Islinger M, Worthy H, Carmichael R, Schrader M. 2024. The peroxisome: an update on mysteries 3.0. Histochem Cell Biol 161:99–132. doi:10.1007/s00418-023-02259-5 38244103 PMC10822820

[23] Brechting PJ, Shah C, Rakotondraibe L, Shen Q, Rappleye CA. 2023. Histoplasma capsulatum requires peroxisomes for multiple virulence functions including siderophore biosynthesis. mBio 14:e0328422. doi:10.1128/mbio.03284-22 37432032 PMC10470777

[24] Choo CYL, Wu P-C, Yago JI, Chung K-R. 2023. The Pex3-mediated peroxisome biogenesis plays a critical role in metabolic biosynthesis, stress response, and pathogenicity in Alternaria alternata. Microbiol Res 266:127236. doi:10.1016/j.micres.2022.127236 36334316

[25] Xu Y, Lu H, Zhu S, Li W-Q, Jiang Y-Y, Berman J, Yang F. 2021. Multifactorial mechanisms of tolerance to ketoconazole in Candida albicans. Microbiol Spectr 9:e0032121. doi:10.1128/spectrum.00321-21 34160280 PMC8552639

[26] Altamirano S, Simmons C, Kozubowski L. 2018. Colony and single cell level analysis of the heterogeneous response of Cryptococcus neoformans to fluconazole. Front Cell Infect Microbiol 8:203. doi:10.3389/fcimb.2018.00203 29971221 PMC6018158

[27] Pettit RK, Repp KK, Hazen KC. 2010. Temperature affects the susceptibility of Cryptococcus neoformans biofilms to antifungal agents. Med Mycol 48:421–426. doi:10.1080/13693780903136879 19637092

[28] Chauhan M, Shivarathri R, Aptekmann AA, Chowdhary A, Kuchler K, Desai JV, Chauhan N. 2025. The Gcn5 lysine acetyltransferase mediates cell wall remodeling, antifungal drug resistance, and virulence of Candida auris. mSphere 10:e0006925. doi:10.1128/msphere.00069-25 40066990 PMC12039264

[29] Lange T, Sprague JL, Vij R, Alonso-Roman R, Jablonowski N, Radosa S, Krüger T, Kniemeyer O, Hillmann F, Brakhage AA, Allert S, Brunke S, Hube B. 2025. “Pour some sugar on me”—Environmental Candida albicans isolates and the evolution of increased pathogenicity and antifungal resistance through sugar adaptation. PLoS Pathog 21:e1013542. doi:10.1371/journal.ppat.1013542 41066457 PMC12510538

[30] Xu D, Wang M, Zhang X, Mao H, Xu H, Zhang B, Zeng X, Li F. 2024. The putative cytochrome b5 domain-containing protein cadap1 homologue is involved in antifungal drug tolerance, cell wall chitin maintenance, and virulence in Candida albicans. J Fungi (Basel) 10:316. doi:10.3390/jof10050316 38786671 PMC11122062

[31] Yang F, Scopel EFC, Li H, Sun L-L, Kawar N, Cao Y-B, Jiang Y-Y, Berman J. 2023. Antifungal tolerance and resistance emerge at distinct drug concentrations and rely upon different aneuploid chromosomes. mBio 14:e0022723. doi:10.1128/mbio.00227-23 36877011 PMC10127634

[32] Zhou X, Hilk A, Solis NV, Pereira De Sa N, Hogan BM, Bierbaum TA, Del Poeta M, Filler SG, Burrack LS, Selmecki A. 2024. Erg251 has complex and pleiotropic effects on sterol composition, azole susceptibility, filamentation, and stress response phenotypes. PLoS Pathog 20:e1012389. doi:10.1371/journal.ppat.1012389 39078851 PMC11315318

[33] Carolus H, Sofras D, Boccarella G, Sephton-Clark P, Biriukov V, Cauldron NC, Lobo Romero C, Vergauwen R, Yazdani S, Pierson S, Jacobs S, Vandecruys P, Wijnants S, Meis JF, Gabaldón T, van den Berg P, Rybak JM, Cuomo CA, Van Dijck P. 2024. Acquired amphotericin B resistance leads to fitness trade-offs that can be mitigated by compensatory evolution in Candida auris. Nat Microbiol 9:3304–3320. doi:10.1038/s41564-024-01854-z 39567662

[34] Massic L, Doorley LA, Jones SJ, Richardson I, Siao DD, Siao L, Dykema P, Hua C, Dykema E, Cuomo CA, Rogers PD, Van Hooser S, Van Hooser JE, Kelly SL, Hess D, Rybak JM, Pandori M. 2025. Acquired amphotericin B resistance attributed to a mutated ERG3 in Candidozyma auris. Antimicrob Agents Chemother 69:e0060125. doi:10.1128/aac.00601-25 40980913 PMC12587534

[35] Asadzadeh M, Alfouzan W, Parker JE, Meis JF, Kelly SL, Joseph L, Ahmad S. 2023. Molecular characterization and sterol profiles identify nonsynonymous mutations in ERG2 as a major mechanism conferring reduced susceptibility to amphotericin B in Candida kefyr. Microbiol Spectr 11:e0147423. doi:10.1128/spectrum.01474-23 37358415 PMC10434000

[36] Skowyra ML, Rapoport TA. 2025. Import mechanism of peroxisomal proteins with an N-terminal signal sequence. Nat Cell Biol 27:948–958. doi:10.1038/s41556-025-01662-5 40346349 PMC12173945

[37] Shukla N, Neal ML, Farré J-C, Mast FD, Sarkar R, Truong L, Simon T, Miller LR, Aitchison JD, Subramani S. 2025. TOR and heat shock response pathways regulate peroxisome biogenesis during proteotoxic stress. Nat Commun 16:10743. doi:10.1038/s41467-025-65776-y 41315397 PMC12663454

[38] Bi Z, Wang T, Wang X, Xu H, Wu Y, Zhao C, Wu Z, Yu J, Zhang L. 2024. FpPEX5 and FpPEX7 are involved in the growth, reproduction, DON toxin production, and pathogenicity in Fusarium pseudograminearum. Int J Biol Macromol 270:132227. doi:10.1016/j.ijbiomac.2024.132227 38734339

[39] Chen R, Lu K, Yang L, Jiang J, Li L. 2024. Peroxin MoPex22 regulates the import of peroxisomal matrix proteins and appressorium-mediated plant infection in Magnaporthe oryzae. J Fungi (Basel) 10:143. doi:10.3390/jof10020143 38392815 PMC10890347

[40] Liu Q, Li D, Bai N, Zhu Y, Yang J. 2023. Peroxin Pex14/17 is required for trap formation, and plays pleiotropic roles in mycelial development, stress response, and secondary metabolism in Arthrobotrys oligospora. mSphere 8:e0001223. doi:10.1128/msphere.00012-23 36786584 PMC10117088

[41] Villa S, Hamideh M, Weinstock A, Qasim MN, Hazbun TR, Sellam A, Hernday AD, Thangamani S. 2020. Transcriptional control of hyphal morphogenesis in Candida albicans. FEMS Yeast Res 20:foaa005. doi:10.1093/femsyr/foaa005 31981355 PMC7000152

[42] Wu Y, Chen Y, Lu H, Ying C. 2023. Miltefosine exhibits fungicidal activity through oxidative stress generation and Aif1 activation in Candida albicans. Int J Antimicrob Agents 62:106819. doi:10.1016/j.ijantimicag.2023.106819 37072087

[43] Gerstein AC, Rosenberg A, Hecht I, Berman J. 2016. diskImageR: quantification of resistance and tolerance to antimicrobial drugs using disk diffusion assays. Microbiology (Reading) 162:1059–1068. doi:10.1099/mic.0.000295 27126388 PMC5756480

[44] Wu Y, Dai Y, Lu H, Jiang X, Wang Y. 2025. Osh2 mediates Candida species resistance to miltefosine by regulating zymosterol accumulation. Antimicrob Agents Chemother 69:e0042725. doi:10.1128/aac.00427-25 40698822 PMC12406662

