# Supplementary figures and images for: Peroxin Pex8 couples stress responses, antifungal tolerance, and virulence regulation in Candida albicans
Source: Antimicrob Agents Chemother. 2026 Mar 24;70(5):e01662-25. doi: 10.1128/aac.01662-25 (PMC13148049; doi:10.1128/aac.01662-25)

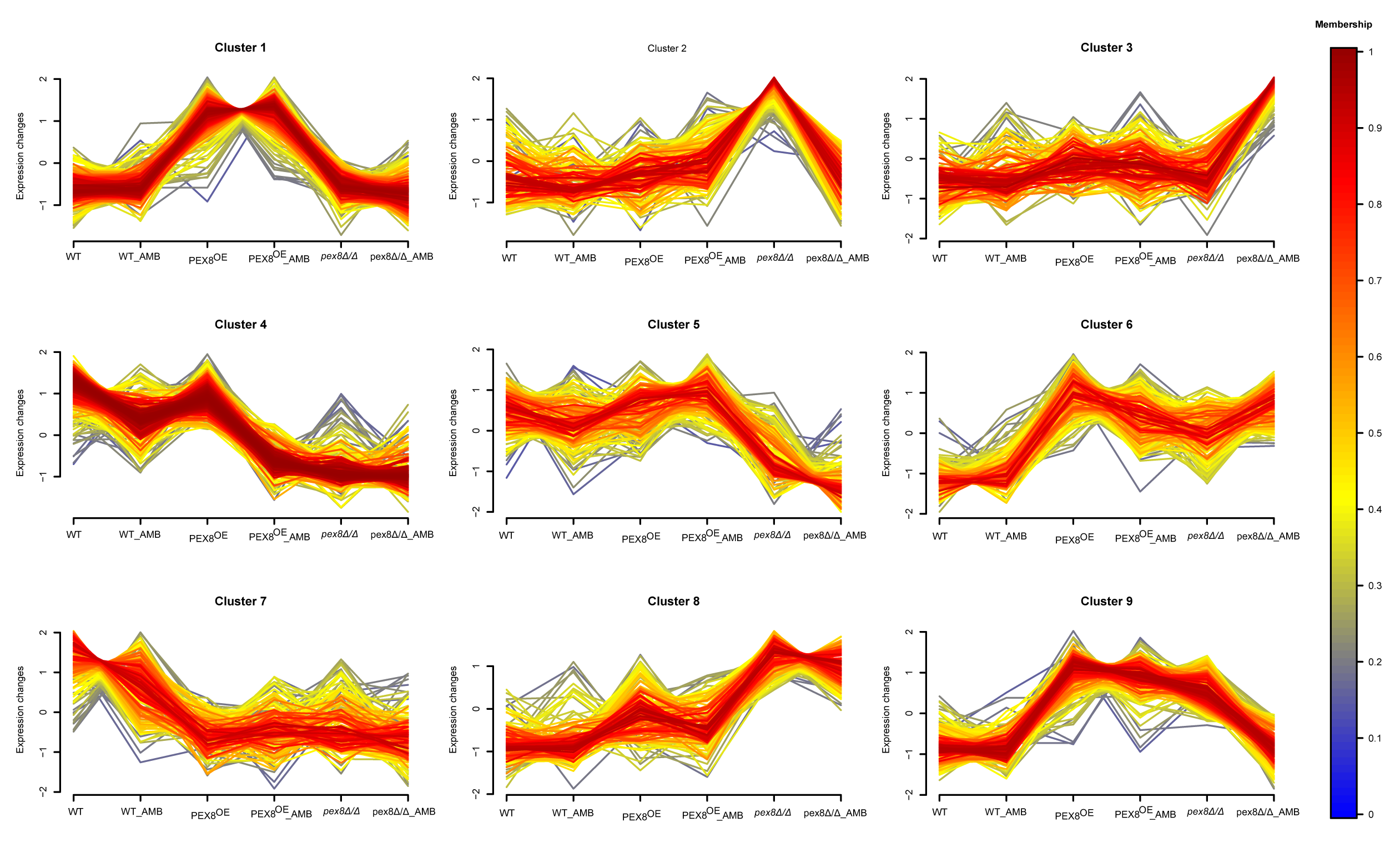

Supplement: Fig. S1 — Trend clustering analysis was employed to elucidate the expression patterns and dynamic changes of all identified lipid molecules across different groups. [file aac.01662-25-s0001.tif]

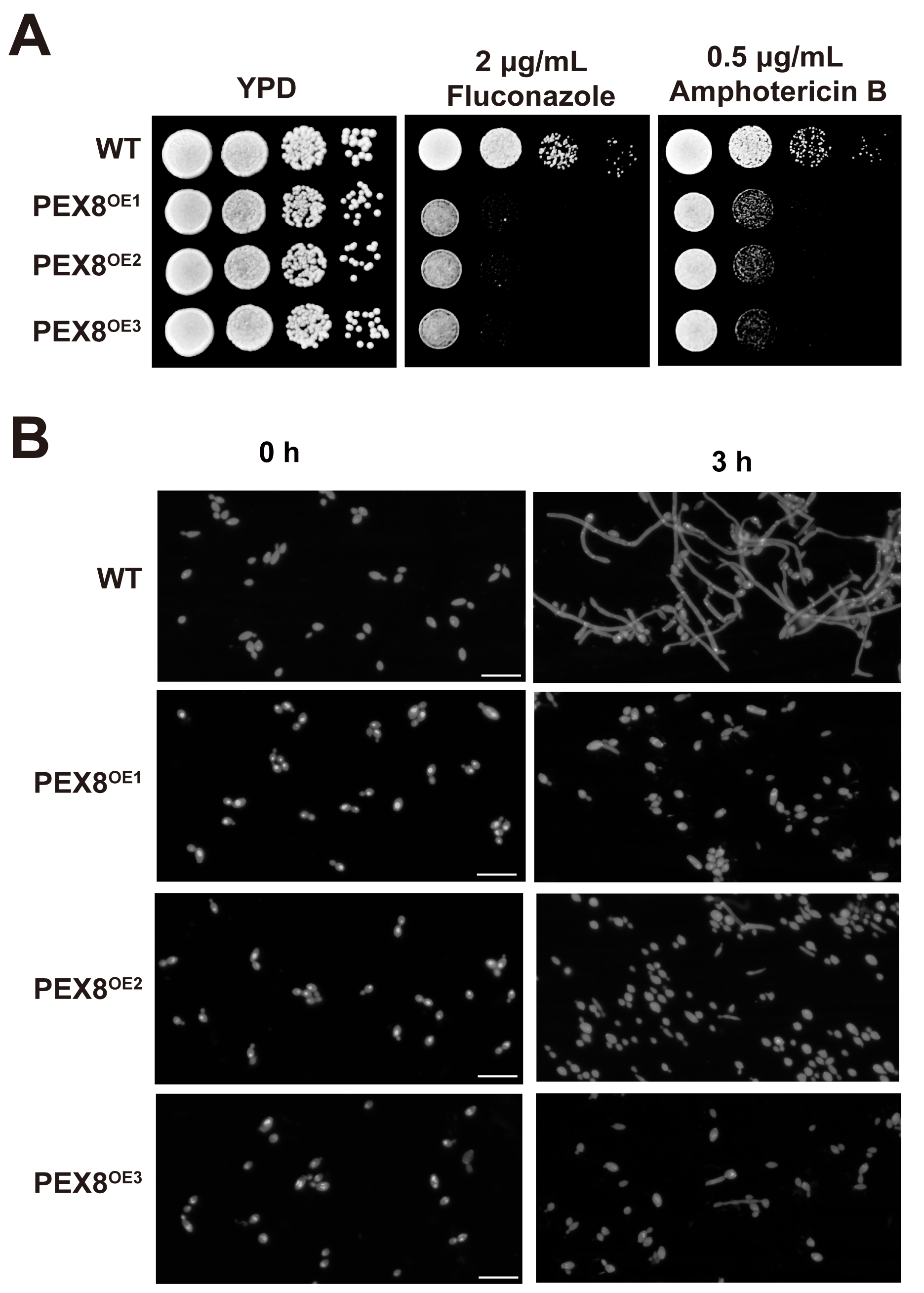

Supplement: Fig. S2 — PEX8 overexpression reduces antifungal drug susceptibility and impairs hyphal morphogenesis. [file aac.01662-25-s0002.tif]
