# Supplementary material for: Peroxin Pex8 couples stress responses, antifungal tolerance, and virulence regulation in Candida albicans
Source: Antimicrob Agents Chemother. 2026 Mar 24;70(5):e01662-25. doi: 10.1128/aac.01662-25 (PMC13148049; doi:10.1128/aac.01662-25)
Supplement: Supplemental material — Supplemental figure and table legends. [file aac.01662-25-s0003.docx]

**Supplemental legends:**

**Figure S1.** Trend clustering analysis was employed to elucidate the expression patterns and dynamic changes of all identified lipid molecules across different groups. Lipid profiles were grouped by fuzzy c-means clustering, partitioning molecules according to their expression trajectories across conditions.

**Figure S2.** *PEX8* overexpression reduces antifungal drug susceptibility and impairs hyphal morphogenesis. (A) Drug susceptibility profiling by spot assay. Overnight cultures of wild‑type (WT) and three independent *PEX8*-overexpressing (*PEX8^OE^*) strains were washed with PBS, adjusted to 10^7^ cells/mL. Ten-fold serial dilutions (5 μL) of these strains were spotted on YPD plates containing the indicated drugs. Plates were incubated at 37°C for 24 h. (B) Serum-induced hyphal formation assay. Strains grown to logarithmic phase were washed twice with PBS and adjusted to an OD_600_ of 1.0. Hyphal induction was performed in YPD medium supplemented with 10% fetal bovine serum at 37 °C with shaking. Samples were collected at 0 and 3 h post‑induction, washed, stained with calcofluor white (CFW), and imaged by fluorescence microscopy. Scale bar, 20 μm.

**Table S1.** Differentially expressed genes analysis.

**Table S2.** Differentially abundant lipid species.

**Table S3.** Strains and plasmids used in this study.

**Table S4.** Primers used in this study.
